# Supplementary material for: Complex‐centric proteome profiling by SEC‐SWATH‐MS
Source: Mol Syst Biol. 2019 Jan 14;15(1):e8438. doi: 10.15252/msb.20188438 (PMC6346213; doi:10.15252/msb.20188438)
Supplement: Supplementary file 8 — Dataset EV7 [file MSB-15-e8438-s008.zip › feature_plots_string/Q01581.pdf]

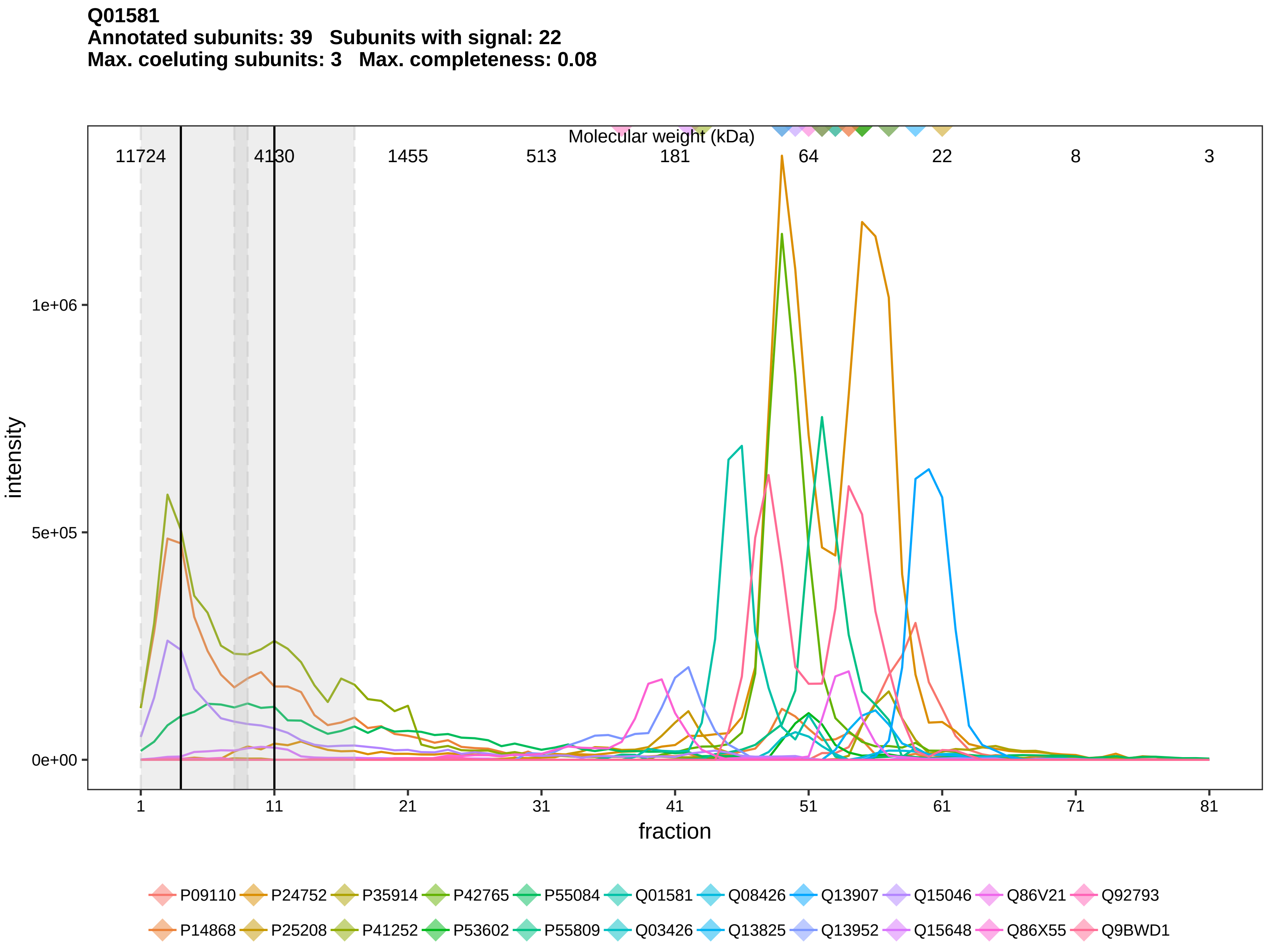

Q01581
Annotated subunits: 39 Subunits with signal: 22
Max. coeluting subunits: 3 Max. completeness: 0.08
Molecular weight (kDa)
11724
4130
1455
513
181
64
22
8
3
intensity
1e+06
5e+05
0e+00
fraction
1
11
21
31
41
51
61
71
81
P09110
P24752
P35914
P42765
P55084
Q01581
Q08426
Q13907
Q15046
Q86V21
Q92793
P14868
P25208
P41252
P53602
P55809
Q03426
Q13825
Q13952
Q15648
Q86X55
Q9BWD1
